# Early life exposure to secondhand tobacco smoke and eating behaviors at age 12 years

**DOI:** 10.1186/s12940-024-01076-0

**Published:** 2024-04-13

**Authors:** Nerea Mourino, Zhuoya Zhang, Mónica Pérez-Ríos, Kimberly Yolton, Bruce P. Lanphear, Aimin Chen, Jessie P. Buckley, Heidi J. Kalkwarf, Kim M. Cecil, Joseph M. Braun

**Affiliations:** 1 Department of Preventive Medicine and Public Health, University of Santiago de Compostela, Santiago de Compostela, Spain; 2 Department of Epidemiology, Dartmouth College, Hanover, NH USA; 3 Consortium for Biomedical Research in Epidemiology and Public Health (CIBERESP), Madrid, Spain; 4 Health Research Institute of Santiago de Compostela (IDIS), Santiago de Compostela, Spain; 5 Department of Pediatrics, Cincinnati Children’s Hospital Medical Center, University of Cincinnati College of Medicine, Cincinnati, OH USA; 6 Faculty of Health Sciences, Simon Fraser University, Vancouver, BC Canada; 7 Department of Biostatistics, Epidemiology and Informatics, Perelman School of Medicine, University of Pennsylvania, Philadelphia, PA USA; 8 Department of Epidemiology, University of North Carolina at Chapel Hill Gillings School of Global Public Health, Chapel Hill, NC USA; 9 Department of Radiology, Cincinnati Children’s Hospital Medical Center, University of Cincinnati College of Medicine, Cincinnati, OH USA; 10 Department of Epidemiology, Brown University, Providence, RI USA

**Keywords:** Secondhand tobacco smoke, Cotinine, Adolescents, Eating behaviors

## Abstract

**Background:**

Prenatal or early childhood secondhand tobacco smoke (SHS) exposure increases obesity risk. However, the potential mechanisms underlying this association are unclear, but obesogenic eating behaviors are one pathway that components of SHS could perturb. Our aim was to assess associations of prenatal and early childhood SHS exposure with adolescent eating behaviors.

**Methods:**

Data came from a prospective pregnancy and birth cohort (*N* = 207, Cincinnati, OH). With multiple informant models, we estimated associations of prenatal (mean of 16 and 26 weeks of gestation maternal serum cotinine concentrations) and early childhood cotinine (average concentration across ages 12, 24, 36, and 48 months) with eating behaviors at age 12 years (Child Eating Behaviors Questionnaire). We tested whether associations differed by exposure periods and adolescent’s sex. Models adjusted for maternal and child covariates.

**Results:**

We found no statistically significant associations between cotinine measures and adolescent’s eating behaviors. Yet, in females, prenatal cotinine was associated with greater food responsiveness (β: 0.23; 95% CI: 0.08, 0.38) and lower satiety responsiveness (β: -0.14; 95% CI: -0.26, -0.02); in males, prenatal and postnatal cotinine was related to lower food responsiveness (prenatal: β: -0.25; 95% CI: -0.04, -0.06; postnatal: β: -0.36; 95% CI: -0.06, -0.11). No significant effect modification by sex or exposure window was found for other eating behaviors.

**Conclusion:**

Prenatal and early childhood SHS exposures were not related to adolescent’s eating behavior in this cohort; however, biological sex may modify these associations.

**Supplementary Information:**

The online version contains supplementary material available at 10.1186/s12940-024-01076-0.

## Introduction

The prevalence of obesity has tripled globally since 1975 and increased dramatically in the last three decades in the United States (US) [1]. In 2017–2020, the prevalence in US adolescents aged 12–19 reached 22% [2]. Obesity is a risk factor for adulthood cardiovascular disease [3] and metabolic syndrome (MetS), a multifactorial disorder characterized by the cluster of central obesity, impaired glucose metabolism, dyslipidemia, and elevated blood pressure [4]. Diminished cardiometabolic health (CM) in childhood increases their risk of all-cause mortality, cardiovascular disease morbidity and mortality, and type 2 diabetes in adulthood [5].

Exposure to secondhand tobacco smoke (SHS) is a risk factor for the development of obesity and CM disorders during adolescence [6, 7]. Evidence suggests maternal behaviors, such as poor nutrition and exposure to chemicals in SHS, can lead to intrauterine growth restriction and, thus, poor CM health in children and adolescents [8, 9], consistent with what we observed in our cohort of 12-year-old adolescents from the Health Outcomes and Measures of the Environment (HOME) Study [6, 7]. In 2022, we found that postnatal SHS exposure was related to higher adiposity and CM risk and that the exposure time window and sex modify the strength of associations [6, 7].

Some chemicals found in SHS, including polycyclic aromatic hydrocarbons and nitrosamine 4 (methylnitrosamino)-1-(3-pyridyl)-1-butanone, can cross the placenta and directly impact hypothalamic neuropeptides and amygdala volume, which play a role in emotional regulation and reward reactivity [10, 11]. Further, nicotine from SHS may affect taste and smell perception [12, 13]. These alterations can influence children’s appetite, food intake, and eating preferences. Therefore, children exposed to SHS during gestation or early childhood may exhibit more obesogenic eating behaviors, such as lower satiety sensitivity, higher food responsiveness, and emotional overeating, and thus, in turn, may develop CM disorders later in life [11, 14].

The role of appetite in obesity risk was recognized in 1968 by Schachter [15]. Wardle and colleagues developed the behavioral susceptibility theory of obesity, which hypothesized that appetite mediates the interaction between genetic susceptibility to obesity and exposure to obesogenic environments and that variation in appetite emerges early in postnatal life [16]. However, whether eating behaviors may act as a behavioral pathway underlying the association between SHS exposure and obesity or CM disorders remains unclear. A better understanding will inform effective preventative strategies and interventions for childhood obesity.

This study examined the association of prenatal and early childhood serum cotinine concentrations with adolescent’s eating behaviors among the HOME Study participants. Based on prior findings from the HOME Study [6, 7], which emphasized the greater impact of postnatal SHS exposure on adolescents’ health outcomes compared to prenatal exposure, with a stronger association observed in girls than in boys, we hypothesized that: (1) Higher serum cotinine concentrations would be associated with greater food approaching behaviors (i.e., food responsiveness and emotional overeating) and lower food avoidance behaviors (i.e., satiety responsiveness and emotional undereating); (2) Associations between serum cotinine and eating behaviors are stronger during the postnatal period, compared to the prenatal period; and (3) Associations between serum cotinine and eating behaviors are stronger in female adolescents than in males.

## Methods

### Study recruitment and data collection

We used data from a prospective and ongoing pregnancy and birth cohort, the HOME Study [17]. The HOME Study recruited pregnant women between 2003 and 2006 and conducted follow-up visits with the mothers and their children through age 12 years. The study aimed to evaluate the association of pre- and postnatal exposure to environmental toxicants with a child’s growth and neurobehavioral outcomes. From 2003 to 2014, we conducted up to 11 in-person follow-up visits on 410 eligible children at the delivery hospital, the study clinic, or participants’ homes from birth to age 12 years. From June 2016 to April 2019, we invited 441 adolescents and their birth mother or primary caregiver to participate in a clinical visit, and 256 adolescents completed this 12-year visit. A detailed description of inclusion criteria, participants’ characteristics, follow-up, and study measures at the 12-year visit are described elsewhere [17]. Our final analytic sample included singleton children without congenital anomalies with at least one serum cotinine concentration measurement during pregnancy or childhood, caregivers’ responses to the Child Eating Behavior Questionnaire (CEBQ), and complete covariate information (*N* = 207).

The institutional review boards (IRBs) at Cincinnati Children’s Hospital Medical Center (CCHMC) and participating delivery hospitals approved the HOME Study protocols. Brown University and the Centers for Disease Control and Prevention (CDC) IRBs deferred to the CCHMC IRB. All mothers provided informed consent for themselves and their children at all visits; children provided informed assent at the age 12 visit.

### Prenatal and postnatal SHS exposure assessment

Trained phlebotomists collected maternal venous blood samples at 16 and 26 weeks of pregnancy to assess prenatal SHS exposure. They also collected the child’s venous blood samples at 12, 24, 36, and 48 months via venipuncture to assess postnatal SHS exposure. The samples were stored at or below − 80ºC until analysis. Serum cotinine concentrations were quantified by trained technicians at the CDC using high-performance liquid chromatography atmospheric pressure tandem mass spectrometry. The assay limit for the cotinine detection threshold was 0.015 ng/mL. Maternal serum cotinine concentrations ≥ 3 ng/mL at 16 or 26 weeks of gestation were considered indicative of maternal tobacco consumption [18]. We imputed cotinine levels of 0 ng/mL with 0.001 before log_10_ transforming cotinine concentrations.

### Adolescent’s eating behavior assessment

At the 12-year study visit, caregivers completed the Child Eating Behaviors Questionnaire (typically mothers) to assess their children’s eating behaviors [19]. The CEBQ is a valid and reliable multi-dimensional parent-reported measure of child eating behaviors used worldwide in pediatric research. The CEBQ comprises 35 Likert-style questions (1: never; 2: rarely; 3: sometimes; 4: often; 5: always) to assess eight eating behavior dimensions [19]. A higher score indicates a greater presence of the behavior perceived by the caregiver. Four dimensions measure food approach behaviors: food responsiveness (child’s reaction to external cues for eating); enjoyment of food (child’s interest in food); emotional overeating (child’s behavior of eating more in response to negative emotions such as anxiety, worry or boredom); desire to drink (child’s frequent need for a drink). Four dimensions measure food avoidance behaviors: satiety responsiveness (child’s ability to stop eating when feeling full); slowness in eating (child’s speed of eating); emotional undereating (child’s behavior of eating less in response to negative emotions such as anger and upset); food fussiness (child’s selectiveness about food) [19]. Further, we summed the scores across food approach (16 items) and food avoidance items (19 items) to summarize overall food approach and food avoidance behaviors.

### Covariate assessment

Based on previous literature and directed acyclic graphs (DAGs), we identified potential confounders that may be associated with both serum cotinine concentrations and eating behaviors while ensuring we did not adjust for mediators or colliders (Figures S1 and S2). Mothers completed standardized questionnaires at baseline and reported the child’s race/ethnicity, maternal age, parity, level of education, household income, and marital status. Mothers reported breastfeeding duration via standardized questionnaires over the first three years after delivery. We extracted the child’s sex data from hospital medical records.

### Statistical analysis

We computed summary statistics of maternal and child characteristics, maternal serum cotinine concentrations at 16 and 26 weeks of pregnancy, and the child’s serum cotinine concentrations at age 12, 24, 36, and 48 months. Additionally, we conducted a bivariate analysis to compare the distribution of adolescents’ eating behaviors at age 12 years across strata of potential confounders.

Of 256 children who had complete CEBQ data, 251 and 232 mothers had at least one serum cotinine measurement at 16 and 26 weeks of pregnancy, and 186, 149, 157, and 127 children had cotinine measurements at age 12, 24, 36, and 48 months, respectively. After examining the distribution of the cotinine concentrations by creating histograms and performing Shapiro-Wilk tests, we decided to log_10_-transform the cotinine concentrations to reduce the influence of outliers. We calculated intraclass correlation coefficients (ICC) with 95% confidence intervals (CI) between log_10_-transformed pre- or postnatal cotinine concentrations at each time point. The ICC between repeated maternal cotinine concentrations (*N* = 193 mothers) was 0.91 (95% CI 0.89 to 0.93); the ICC between the four childhood cotinine concentrations (*N* = 64 children) was 0.79 (95% CI 0.69 to 0.86), indicating excellent agreement. Thus, we averaged available maternal serum cotinine concentrations (range = 1–2 measures) and children’s serum cotinine concentrations (range = 1–4 measures) to assess pre- and postnatal exposure, respectively. Spearman correlation coefficients (rho) were computed to evaluate the correlations between the exposure variables (pre- and postnatal serum cotinine concentrations at each time point, average pre- and postnatal concentrations) and the outcome variables (CEBQ dimension scores, food approach summary score, and food avoidance summary score).

Using multiple informant models, we estimated differences in food approach and avoidance behavior summary scores and individual behavior dimension scores with a 1-unit increase in prenatal and postnatal average log_10_-transformed serum cotinine concentrations (β and 95% CI). We further tested whether the strength of associations differed between the pre- and postnatal periods. Details on the multiple informant method have been previously described [20]. The multiple informant model utilizes generalized estimating equations to jointly evaluate the exposure-outcome association for each defined exposure period and tests whether the estimates for the exposure-outcome association are equal across different time points [20]. We created two separate joint estimates, one for the prenatal exposure period (average maternal serum cotinine concentrations at 16 and 26 weeks of pregnancy) and another for the postnatal exposure period (average child’s serum cotinine concentrations at age 12, 24, 36, and 48 months). The multiple informant model included exposure period, serum cotinine, cotinine × exposure period, and covariates, allowing us to test whether cotinine-outcome associations differed between pre- and postnatal exposure periods. The null hypothesis is that the association is constant regardless of exposure time. We considered a *p*-value of the time-exposure interaction term < 0.05 as evidence that at least one of the cotinine-outcome associations differs over time.

We also examined the potential modifying effects of adolescent sex using a three-way interaction term of exposure period × cotinine × adolescent sex. Multiple informant models were adjusted for maternal age at delivery, parity at baseline, maternal education, household income, marital status, duration of any breastfeeding, and adolescent sex and race/ethnicity.

We performed the analyses using R statistical software, version 4.1.2 (R Core Team, Vienna, Austria).

### Role of the funding source

The funder of the study was not involved in the study design, the collection, analysis, and interpretation of data, the manuscript writing, or the decision to submit the paper for publication.

## Results

The characteristics of our sample (*N* = 207) are similar to the baseline study cohort (*N* = 389) (Supplementary Table 1). The mean age of the 207 adolescents was 12.3 years (SD: 0.7; range 11.0–14.0). Among them, 57% were girls, and 62% were non-Hispanic White (Supplementary Table 1). On average, mothers of these adolescents were 27.8 years (SD: 5.8; range: 18.8–45.1) at delivery, and 43% were nulliparous. At the 12-year visit, 88% had greater than high school education, 69% were married, and 45% fed their children some human breastmilk for at least six months (Table S1).

Compared with the respective referent groups, food approach behavior scores were higher (0.9–1.2 points) among adolescents who were non-Hispanic Black and whose mothers were younger at delivery, had a lower level of education, had lower household income, and were unmarried (Table 1). Although food avoidance behavior scores varied by covariates to a lesser extent, they were higher among adolescents who were non-Hispanic White and whose mothers were older at delivery, multiparous, had higher household income, and breastfed their child for at least six months, compared to other groups (Table 1).

**Table 1:** HOME Study participant’s eating behavior (Child Eating Behavior Questionnaire dimensions) at age 12 years according to covariates (*N* = 207)

		Food Approach Behaviors ^a^	Food Avoidance Behaviors ^b^
	N	Mean ± SD	Mean ± SD
**MATERNAL CHARACTERISTICS**			
**Age at delivery (years)**			
18–25	46	11.0 ± 2.7	9.9 ± 2.3
>25–29	43	11.6 ± 3.0	9.7 ± 2.2
>29–34	74	10.3 ± 2.1	10.2 ± 1.9
>34	44	10.6 ± 2.2	10.3 ± 1.9
**Parity at baseline**			
0	88	10.7 ± 2.5	9.7 ± 2.1
1	68	10.8 ± 2.3	10.2 ± 1.9
1+	51	11.0 ± 2.8	10.4 ± 2.1
**Education at baseline**			
High school or less	41	11.5 ± 2.6	10.3 ± 2.0
Technical school or some college	62	10.8 ± 2.7	9.8 ± 2.0
Bachelor’s degree or more	104	10.5 ± 2.3	10.1 ± 2.1
**Household income at baseline ($)**			
< 45,000	81	11.3 ± 2.8	9.8 ± 2.1
45,000–75,000	71	10.5 ± 2.0	10.1 ± 2.1
> 75,000	55	10.5 ± 2.5	10.2 ± 1.9
**Marital status at baseline**			
Married	138	10.4 ± 2.3	10.1 ± 2.1
Not married, living with partner	21	11.1 ± 2.9	10.1 ± 2.5
Not married, living alone	48	11.6 ± 2.7	9.9 ± 1.9
**Breastfeeding duration (months)**^**c**^			
<6	113	11.0 ± 2.6	9.8 ± 2.0
≥6	94	10.5 ± 2.4	10.3 ± 2.1
**ADOLESCENT’S CHARACTERISTICS**			
**Sex**			
Female	117	11.2 ± 2.5	10.2 ± 2.1
Male	90	10.3 ± 2.5	9.8 ± 1.9
**Race/ethnicity**			
White, non-Hispanic	129	10.5 ± 2.3	10.2 ± 2.0
Black, non-Hispanic	66	11.4 ± 2.8	9.9 ± 2.0
Others	12	10.4 ± 2.6	9.8 ± 2.3

^a^The sum of the scores on the food responsiveness, emotional overeating, enjoyment of food, and desire to drink from the CEBQ dimensions

^b^The sum of the scores on the satiety responsiveness, slowness in eating, emotional undereating, and food fussiness from the CEBQ dimensions

^c^Data was obtained at the 3-year visit

**Fig. 1:**
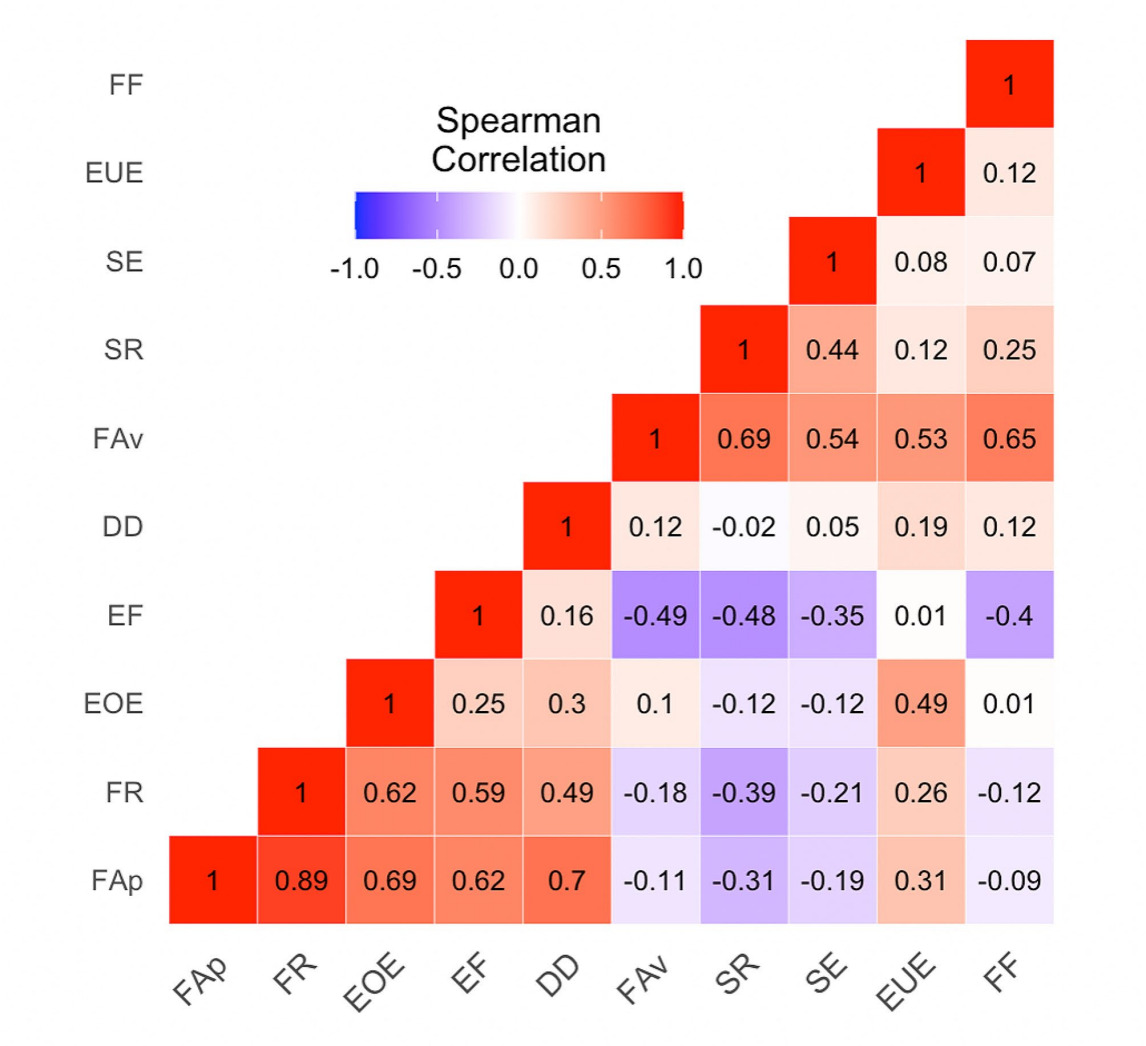
Spearman correlation coefficients between Child Eating Behavior Questionnaire (CEBQ) dimensions among HOME Study adolescents (*N* = 207). *Abbreviations*: FAp, food approach summary score; FR, food responsiveness; EOE, emotional overeating; EF, enjoyment of food; DD, desire to drink; FAv, food avoidance summary score; SR, satiety responsiveness; SE, slowness in eating; EUE, emotional

Female adolescents scored 0.9 points higher on the food approach summary score than males (Table 2). Considering the four food approach behavior dimensions, females generally scored 0.3 points higher on emotional overeating and desire to drink than males. Regarding food avoidance summary scores, females scored 0.4 points higher than males, with the greatest increase being for emotional undereating (0.2 points) (Table 2). Eating behavior measures at age 12 years were weakly to strongly correlated with each other, with coefficients ranging from 0.01 (enjoyment of food, emotional undereating) to 0.62 (food responsiveness, emotional overeating) (Fig. 1).

**Table 2:** Descriptive statistics of Child Eating Behavior Questionnaire (CEBQ) dimensions in the HOME Study participants at age 12 years and stratified by adolescent sex (*N* = 207)

Eating Behaviors Subscale	All (*N* = 207)	Females (*N* = 117)	Males (*N* = 90)
	Mean ± SD	Mean ± SD	Mean ± SD
**Food Approach Summary Score**	10.8 ± 2.5	11.2 ± 2.5	10.3 ± 2.5
Food responsiveness	2.4 ± 0.9	2.5 ± 0.9	2.3 ± 0.8
Emotional overeating	2.0 ± 0.8	2.1 ± 0.8	1.8 ± 0.7
Enjoyment of food	4.0 ± 0.7	4.0 ± 0.7	3.9 ± 0.8
Desire to drink	2.4 ± 1.0	2.6 ± 1.1	2.3 ± 1.0
**Food Avoidance Summary Score**	10.0 ± 2.1	10.2 ± 2.1	9.8 ± 1.9
Satiety responsiveness	2.6 ± 0.7	2.6 ± 0.7	2.5 ± 0.7
Slowness in eating	2.4 ± 0.7	2.3 ± 0.7	2.4 ± 0.8
Emotional undereating	2.5 ± 0.9	2.6 ± 0.8	2.4 ± 1.0
Food fussiness	2.6 ± 0.9	2.7 ± 0.9	2.6 ± 1.0

Approximately 9% of mothers (*N* = 19) had average serum cotinine concentrations indicative of active smoking during pregnancy (≥ 3ng/mL). Median prenatal serum cotinine concentrations were similar to median postnatal concentrations (0.03 vs. 0.05 ng/mL, respectively) (Table S2). Median pre- and postnatal cotinine concentrations were similar between females and males (0.04 vs. 0.02 ng/mL and 0.06 vs. 0.05 ng/mL, respectively, with *p*-values > 0.05 via two-sample t-tests) (Table S2 and Figure S3). Postnatal serum cotinine concentrations were moderately correlated with the prenatal concentrations (rho = 0.79) (Figure S4).

After adjusting for covariates, we generally did not observe notable associations of prenatal or postnatal serum cotinine with CEBQ scores (Table 3). Moreover, associations of serum cotinine concentrations with eating behaviors at age 12 years did not differ by exposure periods (cotinine x exposure period interaction terms > 0.05) (Table 3). Results from the multiple informant models reflect no main effects of serum cotinine concentrations on adolescents’ eating behaviors (Table 3).

**Table 3:** Unadjusted and adjusted difference in Child Eating Behavior Questionnaire (CEBQ) dimensions (β with 95% CI) per log_10_-transformed increase in prenatal and postnatal serum cotinine concentrations (*N* = 207)

	UNADJUSTED MODEL					ADJUSTED MODEL^a^				
	**Prenatal**		**Postnatal**			**Prenatal**		**Postnatal**		
**Outcome**	**β**	**95% CI**	**β**	**95% CI**	**Interaction** ***p*** **-value** ^**b**^	**β**	**95% CI**	**β**	**95% CI**	**Interaction** ***p*** **-value** ^**b**^
**Food Approach Summary Score**	0.45	0.13, 0.78	0.53	0.12, 0.94	0.78	0.11	-0.23, 0.46	-0.10	-0.59, 0.39	0.40
Food responsiveness	0.13	0.01, 0.25	0.14	-0.01, 0.28	0.95	0.02	-0.11, 0.15	0.06	-0.25, 0.12	0.37
Emotional overeating	0.01	-0.10, 0.10	0.02	-0.11, 0.14	0.96	0.02	-0.07, 0.12	0.03	-0.13, 0.18	0.98
Enjoyment of food	0.04	-0.04, 0.12	-0.01	-0.14, 0.12	0.49	0.00	-0.09, 0.10	-0.07	-0.23, 0.10	0.36
Desire to drink	0.27	0.12, 0.42	0.38	0.22, 0.55	0.30	0.06	-0.09, 0.21	0.01	-0.19, 0.20	0.58
**Food Avoidance Summary Score**	-0.15	-0.41, 0.10	-0.13	-0.49, 0.23	0.90	-0.17	-0.48, 0.14	-0.06	-0.53, 0.40	0.63
Satiety responsiveness	-0.13	-0.20, -0.06	-0.15	-0.26, -0.03	0.77	-0.06	-0.16, 0.03	0.00	-0.16, 0.20	0.38
Slowness in eating	0.00	-0.07, 0.07	0.02	-0.09, 0.14	0.73	-0.06	-0.14, 0.02	-0.05	-0.20, 0.10	0.91
Emotional undereating	-0.01	-0.12, 0.11	0.00	-0.15, 0.16	0.90	0.00	-0.14, 0.13	0.00	-0.18, 0.19	0.94
Food fussiness	-0.02	-0.14, 0.10	-0.01	-0.16, 0.14	0.91	-0.04	-0.19, 0.10	-0.02	-0.22, 0.19	0.78

^a^Models adjusted for maternal age at delivery, parity at baseline, education, household income, marital status, duration of any breastfeeding, and adolescent’s sex, and race/ethnicity

^b^From the two-way interaction term between exposure period and serum cotinine

The three-way interaction term of cotinine × exposure period × adolescent’s sex in the multiple informant models was not statistically significant (*p*-value for interaction > 0.05) (Table 4). However, higher prenatal cotinine concentrations were associated with more food approach in females but not in males. Specifically, each 1-unit increase in prenatal log_10_-transformed cotinine concentrations was associated with greater food responsiveness (β: 0.23; 95%CI: 0.08, 0.38) and lower satiety responsiveness (β: -0.14; 95%CI: -0.26, -0.02) among females (Table 4). Of note, each 10-fold increase in cotinine concentrations was associated with lower food responsiveness in males (prenatal β: -0.25; 95%CI: -0.44, -0.06; postnatal β: -0.36; 95%CI: -0.60, -0.11) (Table 4).

**Table 4:** Adjusted difference in Child Eating Behavior Questionnaire (CEBQ) dimension scores (β with 95% CI) per log_10_-transformed increase in prenatal and postnatal serum cotinine concentrations (*N* = 207): Stratified by adolescent’s sex

	Prenatal				Postnatal				
	Females (*N* = 117)		Males (*N* = 90)		Females (*N* = 117)		Males (*N* = 90)		Interaction *p*-value^b^
Outcome	β ^a^	95% CI	β	95% CI	β	95% CI	β	95% CI	
**Food Approach Summary Score**	0.56	0.16, 0.97	**-**0.45	-0.84, -0.06	0.44	-0.20, 1.09	-0.64	-1.32, 0.04	0.93
Food responsiveness	0.23	0.08, 0.38	-0.25	-0.44, -0.06	0.19	-0.05, 0.44	-0.36	-0.60, -0.11	0.86
Emotional overeating	0.10	-0.03, 0.22	-0.04	-0.17, 0.09	0.11	-0.10, 0.33	-0.03	-0.23, 0.16	0.95
Enjoyment of food	0.07	-0.04, 0.19	-0.08	-0.23, 0.08	0.08	-0.11, 0.28	-0.22	-0.48, 0.03	0.43
Desire to drink	0.17	-0.01, 0.35	-0.08	-0.26, 0.10	0.05	-0.20, 0.30	-0.03	-0.32, 0.27	0.33
**Food Avoidance Summary Score**	-0.18	-0.62, 0.25	-0.08	-0.49, 0.33	-0.11	-0.75, 0.54	0.05	-0.60, 0.70	0.92
Satiety responsiveness	-0.14	-0.26, -0.02	0.01	-0.12, 0.15	-0.07	-0.29, 0.14	0.04	-0.17, 0.25	0.82
Slowness in eating	-0.07	-0.16, 0.02	0.01	-0.11, 0.13	-0.06	-0.23, 0.11	-0.04	-0.28, 0.19	0.63
Emotional undereating	0.14	-0.02, 0.30	-0.15	-0.34, 0.04	0.15	-0.09, 0.39	-0.06	-0.32, 0.20	0.64
Food fussiness	-0.11	-0.31, 0.08	0.04	-0.15, 0.24	-0.12	-0.39, 0.16	0.12	-0.18, 0.42	0.76

^a^Beta coefficients derived from multiple informant models adjusting for maternal age at delivery, parity at baseline, education, household income, marital status, duration of any breastfeeding, and adolescent’s sex, and race/ethnicity

^b^From the three-way interaction term of exposure period × serum cotinine × adolescent’s sex

## Discussion

To our knowledge, this is the first study to assess the associations of repeated measures of serum cotinine concentrations from pregnancy to age four years with eating behaviors at age 12 years. We did not find any statistically significant associations between cotinine and eating behaviors or evidence that associations differed by exposure period (prenatal vs. postnatal). However, there were some notable sex-specific associations; prenatal serum cotinine concentrations were associated with higher food responsiveness and lower satiety responsiveness among females aged 12.

Consistent with prior literature that solely assessed prenatal SHS exposure, we did not find any statistically significant associations between prenatal exposure and individual eating behavior dimensions [21]. We are aware of three studies [21–23] that assessed the association between at least one dimension of an infant’s or child’s eating behavior and their prenatal SHS exposure, albeit only with maternal smoking during pregnancy. These studies [21–23] assessed the association of maternal smoking during pregnancy with a child’s eating behavior using subjective methods such as maternal self-reported questionnaires. Consequently, estimates could be inaccurate due to subjectivity linked to differences in perception, ignorance of SHS exposures, or recall and social desirability biases. In addition, the authors adjusted the analysis models for potential casual intermediates (e.g., birth weight or gestational age), which could have resulted in an underestimation of the total effect of prenatal SHS exposure on a child’s eating behaviors. Finally, prior studies did not evaluate postnatal exposure or potential sex differences in the impact of prenatal SHS exposure on eating behavior [21–23].

Cardona Cano et al. [23] reported that prenatal SHS exposure among Dutch children was not a determinant of their trajectories of picky eating, as measured by two items (“child refuses to eat” and “child doesn’t eat well”) from the Child Behavior Checklist. This validated parent-report questionnaire assesses children’s emotional and behavioral problems [24]. In contrast to this finding, in 2023, Bourne et al. [22] observed a greater risk of persistent picky eating among Scottish children whose mothers reported smoking during pregnancy (RR = 2.18; 95% CI: 1.34–3.57). However, this study [22] may have been subject to information bias since picky eating was defined based on previous studies rather than validated questionnaires [22]. Picky eating (i.e., fussy, faddy, or selective eating) can occur in early childhood. It may precede the onset of eating disorders in adolescence, such as anorexia or bulimia nervosa [25]. Results from Bourne et al. and Cardona Cano et al. [22, 23] suggest that picky eating may vary over time. In our study, the associations between cotinine and CEBQ-measured food fussiness were null; unfortunately, we did not have repeated measures of individual eating behavior dimensions from childhood to adolescence, which would have allowed us to confirm the above conjecture.

Costa et al. [21] examined the stability of appetitive traits during childhood and their association with prenatal exposure to SHS. This study suggested age-related variation in the associations between prenatal self-reported SHS exposure and some eating behavior dimensions measured by the Baby Eating Behavior Questionnaire. Although associations of prenatal SHS exposure with food responsiveness, food enjoyment, and slowness in eating were null, infants whose mothers self-reported smoking during pregnancy had lower satiety responsiveness at age three months compared to those unexposed (β = − 0.26; 95% CI: − 0.49, − 0.04); however, this association could be biased since authors did not adjust for any maternal sociodemographic characteristics. In our study, we observed that adjustment for maternal age, educational level, income, marital status, parity, duration of breastfeeding, and ethnicity attenuated the association of prenatal cotinine with food responsiveness and satiety responsiveness at age 12 years, suggesting that multiple maternal factors may confound these associations.

The extent to which eating behaviors may manifest differently between females and males, especially in childhood or adolescence, remains unclear. In our cohort, we noticed a potential sex-specific association of maternal SHS exposure on certain appetitive traits in the offspring. For instance, in our sample, prenatal cotinine exposure showed a positive association with food responsiveness (β: 0.23; 95% CI: 0.08, 0.38) in females yet a negative association in males (β: -0.25; 95% CI: -0.04, -0.06). A mini-review of retrospective and prospective cohort studies postulates that pre-natal nicotine exposure may affect offspring’s neurocognitive and behavioral outcomes differently by child’s sex [26]. Yet, findings on the sex differences in maternal smoking-related behavioral outcomes are not always consistent [26]. Further, given the primarily null results in our main analysis, we cannot rule out the possibility of chance findings. Results from the sex-stratified analysis of ours and others highlight the complexity of these associations and possible sex-specific impacts of maternal smoking and children’s behaviors. Additional research is thus needed to confirm if the associations between maternal SHS and offspring’s eating behaviors are sex-specific and what underlying mechanisms may potentially explain this sex effect modification.

Prenatal and early postnatal exposure to SHS can negatively affect the neuroendocrine system in adolescence or adulthood in both females and males, which may increase food preferences for fats and the risk of developing obesity [7, 27, 28]. Studies in rodents have suggested that exposure to maternal smoking during pregnancy or lactation may cause hormonal dysfunction in offspring and alterations in their hypothalamic circuitry involved in appetite control; this may affect the expression patterns of neuropeptides released by leptin-regulated anorexigenic or orexigenic neurons and cause sustained activation of glial cells (such as astrocytes and microglia), which may lead to hypothalamic inflammation [28]. These changes may contribute to alterations in appetite and metabolic control, primarily due to leptin resistance, which has been associated with decreased energy expenditure and satiety responsiveness and increased food responsiveness, both of which are associated with higher adiposity and risk for CM disorders [13, 14]. Moreover, previous studies in humans have found that prenatal exposure to SHS is associated with higher postnatal plasma levels of the appetite-stimulating hormone ghrelin [29], which could lead to increased food responsiveness [30–32]. Ghrelin, an orexigenic hormone, triggers appetite and food-seeking behaviors by activating brain regions associated with hunger and reward [31]. By interacting with neurotransmitters such as dopamine, opioids, endocannabinoids, and orexins, ghrelin increases the urge to eat [30]. This interaction occurs in brain regions such as the hypothalamus and other areas involved in reward processing, where ghrelin may increase the salience of food cues, making food more appealing and potentially leading to an increased response to food stimuli [30, 31]. Future human studies should investigate the possible biological mechanisms of the effect of early childhood SHS exposure on dietary intake during adolescence or adulthood.

This study has some limitations and strengths. First, our modest sample size might limit our ability to identify distinct periods of heightened susceptibility and sex-specific cotinine-outcome associations. Still, no studies have assessed the effects of SHS, measured with serum cotinine during pregnancy and early childhood, on adolescents’ eating behaviors. We did not have serum cotinine measures after age four years, yet these might be more important than earlier measures. Notably, the prospective design of our study enabled us to establish clear temporal relations between SHS exposures and eating behaviors. Attrition in follow-up is another potential limitation. Still, measured sociodemographic characteristics were not substantially different among participants who did and did not complete follow-up at 12 years. The maternal self-report of their offspring’s eating behaviors may contain measurement errors due to subjectivity, ignorance about adolescents’ eating outside the home, and social desirability bias. Yet, the CEBQ has demonstrated good internal consistency in pediatric populations and good correspondence with objective behavioral measures of eating [19]. Although we did not include the father’s report, studies have shown that mothers spend significantly more time than fathers in direct interactions with their children across several familial situations [33]. There is potential residual confounding, such as maternal lifestyle; mothers who did not smoke during pregnancy may be more health conscious, and that might impact their children’s eating behaviors. Another limitation of this study is the potential influence of unaccounted-for dietary nicotine exposure [34]. While nicotine from dietary sources can be a source of cotinine in the blood, the typically low quantities consumed by young children are unlikely to impact measured levels significantly. Future assessments of SHS exposure in children should integrate detailed dietary data and refined serum cotinine cut-points to enhance understanding of exposure patterns. This approach would enable differentiation between significant exposures and minor or accidental exposures, including those resulting from dietary sources of nicotine. In addition, our results should be interpreted with caution, as spurious associations may arise from multiple testing. Finally, our findings may not be generalizable to other populations; reassuringly, serum cotinine concentrations among HOME Study participants were similar to those of other pregnant women and children in the US during enrollment and follow-up [35].

## Conclusions

Prenatal and early childhood serum cotinine concentrations were unrelated to adolescents’ eating behavior in this study. However, prenatal exposure to SHS may be related to hyperphagic behaviors, including lower satiety responsiveness and lower food responsiveness, in female adolescents as compared to males. Future cohorts should examine the magnitude of the effect of SHS on eating behaviors from infancy to adolescence, given its impact on body composition and CM risk components, while considering the influence of the period of exposure, biological sex, and social-familial factors.

### Electronic supplementary material

Below is the link to the electronic supplementary material.

Supplementary Material 1

## Data Availability

Data are available upon reasonable request.

## Abbreviations

CCHMC: Cincinnati Children’s Hospital Medical Center
ICC: Intraclass correlation coefficients
CEBQ: Child Eating Behavior Questionnaire
CDC: Centers for Disease Control and Prevention
CI: Confidence interval
CM: Cardiometabolic
DAGs: Directed acyclic graphs
IRBs: Institutional Review Boards HOME: Health Outcomes and Measures of the Environment
MetS: metabolic syndrome
SHS: Secondhand tobacco smoke
US: United States

## References

[1] Obesity and overweight: World Health Organization. 2021 [ https://www.who.int/news-room/fact-sheets/detail/obesity-and-overweight.

[2] Stierman B, Afful J, Carroll MD, Chen T-C, Davy O, Fink S et al. National Health and Nutrition Examination Survey 2017–March 2020 Prepandemic Data Files Development of Files and Prevalence Estimates for Selected Health Outcomes. 2021. 10.15620/cdc:106273 PMC11513744 39380201

[3] Jacobs DR, Woo JG, Sinaiko AR, Daniels SR, Ikonen J, Juonala M (2022). Childhood Cardiovascular Risk factors and Adult Cardiovascular events. N Engl J Med.

[4] Grundy SM, Brewer HB Jr., Cleeman JI, Smith SC Jr., Lenfant C. Definition of metabolic syndrome: Report of the National Heart, Lung, and Blood Institute/American Heart Association conference on scientific issues related to definition. Circulation. 2004;109(3):433-8. 10.1161/01.CIR.0000111245.75752.C6 14744958

[5] Ford ES (2005). Risks for all-cause mortality, cardiovascular disease, and diabetes associated with the metabolic syndrome: a summary of the evidence. Diabetes Care.

[6] Mourino N, Pérez-Ríos M, Yolton K, Lanphear BP, Chen A, Buckley JP (2023). Pre- and postnatal exposure to secondhand tobacco smoke and cardiometabolic risk at 12 years: periods of susceptibility. Environ Res.

[7] Mourino N, Pérez-Ríos M, Yolton K, Lanphear BP, Chen A, Buckley JP (2022). Pre- and postnatal exposure to secondhand tobacco smoke and body composition at 12 years: periods of susceptibility. Obes (Silver Spring).

[8] Grun F, Blumberg B (2009). Endocrine disrupters as obesogens. Mol Cell Endocrinol.

[9] Lv X, Sun J, Bi Y, Xu M, Lu J, Zhao L (2015). Risk of all-cause mortality and cardiovascular disease associated with secondhand smoke exposure: a systematic review and meta-analysis. Int J Cardiol.

[10] Williams G, Harrold JA, Cutler DJ (2000). The hypothalamus and the regulation of energy homeostasis: lifting the lid on a black box. Proc Nutr Soc.

[11] Haghighi A, Schwartz DH, Abrahamowicz M, Leonard GT, Perron M, Richer L (2013). Prenatal exposure to maternal cigarette smoking, amygdala volume, and fat intake in adolescence. JAMA Psychiatry.

[12] Da Ré AF, Gurgel LG, Buffon G, Moura WER, Marques Vidor DCG, Maahs MAP (2018). Tobacco influence on taste and smell: systematic review of the literature. Int Arch Otorhinolaryngol.

[13] Jo YH, Talmage DA, Role LW (2002). Nicotinic receptor-mediated effects on appetite and food intake. J Neurobiol.

[14] Zhang Z, Li N, Buckley JP, Cecil KM, Chen A, Eaton CB (2023). Associations between eating behaviours and cardiometabolic risk among adolescents in the Health outcomes and measures of the Environment study. Pediatr Obes.

[15] Schachter S (1968). Obesity and eating. Internal and external cues differentially affect the eating behavior of obese and normal subjects. Science.

[16] Llewellyn C, Wardle J (2015). Behavioral susceptibility to obesity: gene-environment interplay in the development of weight. Physiol Behav.

[17] Braun JM, Buckley JP, Cecil KM, Chen A, Kalkwarf HJ, Lanphear BP (2020). Adolescent follow-up in the Health outcomes and measures of the Environment (HOME) study: cohort profile. BMJ Open.

[18] Benowitz NL, Bernert JT, Caraballo RS, Holiday DB, Wang J (2009). Optimal serum cotinine levels for distinguishing cigarette smokers and nonsmokers within different racial/ethnic groups in the United States between 1999 and 2004. Am J Epidemiol.

[19] Wardle J, Guthrie CA, Sanderson S, Rapoport L (2001). Development of the children’s eating Behaviour Questionnaire. J Child Psychol Psychiatry.

[20] Sánchez BN, Hu H, Litman HJ, Téllez-Rojo MM (2011). Statistical methods to study timing of vulnerability with sparsely sampled data on environmental toxicants. Environ Health Perspect.

[21] Costa A, Warkentin S, Ribeiro C, Severo M, Ramos E, Hetherington M (2023). Early life exposures are associated with appetitive traits in infancy: findings from the BiTwin cohort. Eur J Nutr.

[22] Bourne L, Bryant-Waugh R, Mandy W, Solmi F (2023). Investigating the prevalence and risk factors of picky eating in a birth cohort study. Eat Behav.

[23] Cardona Cano S, Tiemeier H, Van Hoeken D, Tharner A, Jaddoe VW, Hofman A (2015). Trajectories of picky eating during childhood: a general population study. Int J Eat Disord.

[24] Mansolf M, Blackwell CK, Cummings P, Choi S, Cella D (2022). Linking the child behavior checklist to the strengths and difficulties Questionnaire. Psychol Assess.

[25] Marchi M, Cohen P (1990). Early childhood eating behaviors and adolescent eating disorders. J Am Acad Child Adolesc Psychiatry.

[26] Sikic A, Frie JA, Khokhar JY, Murray JE (2022). Sex differences in the Behavioural Outcomes of Prenatal Nicotine and Tobacco exposure. Front Neurosci.

[27] Fourth Report on Human Exposure to Environmental Chemicals, Tables U. March, (2021). Atlanta, GA: U.S.: Department of Health and Human Services, Centers for Disease Control and Prevention; 2021.

[28] Peixoto TC, Moura EG, Oliveira E, Younes-Rapozo V, Soares PN, Rodrigues VST (2019). Hypothalamic neuropeptides expression and hypothalamic inflammation in adult rats that were exposed to Tobacco smoke during breastfeeding: sex-related differences. Neuroscience.

[29] Paslakis G, Buchmann AF, Westphal S, Banaschewski T, Hohm E, Zimmermann US (2014). Intrauterine exposure to cigarette smoke is associated with increased ghrelin concentrations in adulthood. Neuroendocrinology.

[30] Di Micioni E, Botticelli L, Del Bello F, Giorgioni G, Piergentili A, Quaglia W (2021). Assessing the role of ghrelin and the enzyme ghrelin O-acyltransferase (GOAT) system in food reward, food motivation, and binge eating behavior. Pharmacol Res.

[31] Perello M, Dickson SL (2015). Ghrelin signalling on food reward: a salient link between the gut and the mesolimbic system. J Neuroendocrinol.

[32] Depoortere I (2009). Targeting the ghrelin receptor to regulate food intake. Regul Pept.

[33] Scaglioni S, Salvioni M, Galimberti C (2008). Influence of parental attitudes in the development of children eating behaviour. Br J Nutr.

[34] Benowitz NL (1996). Cotinine as a biomarker of environmental tobacco smoke exposure. Epidemiol Rev.

[35] Woodruff TJ, Zota AR, Schwartz JM (2011). Environmental chemicals in pregnant women in the United States: NHANES 2003–2004. Environ Health Perspect.

