# Supplementary material for: Early life exposure to secondhand tobacco smoke and eating behaviors at age 12 years
Source: Environ Health. 2024 Apr 13;23:37. doi: 10.1186/s12940-024-01076-0 (PMC11015554; doi:10.1186/s12940-024-01076-0)

Supplementary Material

Early Life Exposure to Secondhand Tobacco Smoke and Eating Behaviors at Age 12 Years

Nerea Mourino (1); Zhuoya Zhang (2); Mónica Pérez-Ríos (1, 3-4); Kimberly Yolton (5); Bruce P Lanphear (6); Aimin Chen (7); Jessie P. Buckley (8); Heidi J. Kalkwarf (5); Kim M. Cecil (5, 9); Joseph M Braun (10).

Table S1. Characteristics of the total HOME Study participants at baseline (N=389 mothers and children) and those who were included in the final analysis (N= 207).

	HOME baseline sample N=389 N (%)	Our analysis sample N=207 N (%)
**MATERNAL CHARACTERISTICS**		
**Age at delivery (years)**		
18-25	96 (24.68)	46 (22.22)
>25-29	80 (20.57)	43 (20.77)
>29-34	133 (34.19)	74 (35.75)
>34	80 (20.57)	44 (21.26)
**Parity at baseline**		
0	171 (43.96)	88 (42.51)
1	124 (31.88)	68 (32.85)
1+	92 (23.65)	51 (24.64)
Missing	2 (0.51)	
**Education at baseline**		
High school or less	95 (24.42)	41 (19.80)
Technical school or some college	98 (25.19)	62 (29.95)
Bachelor’s or more	191 (49.10)	104 (50.24)
Missing	5 (1.29)	
**Household income ($) at baseline**		
< 45,000	153 (39.33)	81 (39.13)
45,000-75,000	133 (34.19)	71 (34.30)
> 75,000	103 (26.48)	55 (26.57)
**Marital status at baseline visit**		
Married	248 (63.75)	138 (66.67)
Not married, living with partner	56 (14.40)	21 (10.14)
Not married, living alone	80 (20.57)	48 (23.19)
Missing	5 (1.29)	
**Breastfeeding duration (months)[a]**		
<6	216 (55.53)	113 (54.59)
≥6	157 (40.36)	94 (45.41)
Missing	16 (4.11)	
**ADOLESCENT’S CHARACTERISTICS**		
**Sex**		
Female	208 (53.47)	117 (56.52)
Male	181 (46.53)	90 (43.48)
**Race/ethnicity**		
White, non-Hispanic	238 (61.18)	129 (62.32)
Black, non-Hispanic	123 (31.62)	66 (31.88)
All others	25 (6.43)	12 (5.8)
Missing	3 (0.77)	

[a]Data obtained at the 3-year visit

Table S2. Distribution of serum cotinine concentrations (ng/mL) from 16-week pregnancy to age 48 months (N=207).

Cotinine concentrations (ng/mL)[a]	Overall		Females		Males		
	N	Median (P25 - P75)	N	Median (P25 - P75)	N	Median (P25 - P75)	p^c
**Average Prenatal**	207[b]	0.03 (0.01 - 0.16)	117	0.04 (0.01 - 0.15)	90	0.02 (0.01 - 0.16)	0.13
16W gestation	202	0.03 (0.01 - 0.16)	115	0.04 (0.01 - 0.17)	87	0.03 (0.01 - 0.16)	0.97
26W gestation	198	0.03 (0.01 - 0.13)	109	0.03 (0.01 - 0.13)	89	0.02 (0.01 - 0.11)	0.68
**Average Postnatal**	207	0.05 (0.02 - 0.59)	117	0.06 (0.02 - 0.82)	90	0.05 (0.02 - 0.30)	0.13
12 months	178	0.06 (0.03 - 0.39)	100	0.06 (0.03 - 0.49)	78	0.06 (0.03 - 0.29)	0.91
24 months	140	0.05 (0.03 - 0.26)	77	0.06 (0.03 - 0.54)	63	0.05 (0.03 - 0.16)	0.11
36 months	146	0.03 (0.02 - 0.21)	82	0.04 (0.02 - 0.62)	64	0.03 (0.02 - 0.09)	0.79
48 months	123	0.03 (0.02 - 0.26)	69	0.04 (0.02 - 0.36)	54	0.03 (0.02 - 0.17)	0.21

[a]Prenatal is average of maternal serum cotinine concentrations in 16 and 26 weeks of pregnancy and newborns cord blood cotinine concentrations within 48 hours of delivery. Postnatal is average of child serum cotinine concentrations across 12-, 24-, 36-, and 48-month samples.
[b]19 mothers (9.17%) had serum cotinine indicative of active smoking (≥ 3ng/mL).

[c]P-values from Welch’s two-sample t-tests to examine differences by child sex.

Figure S1. Directed acyclical graph of prenatal exposure to SHS and eating behaviors at age 12 years considering potential confounders.

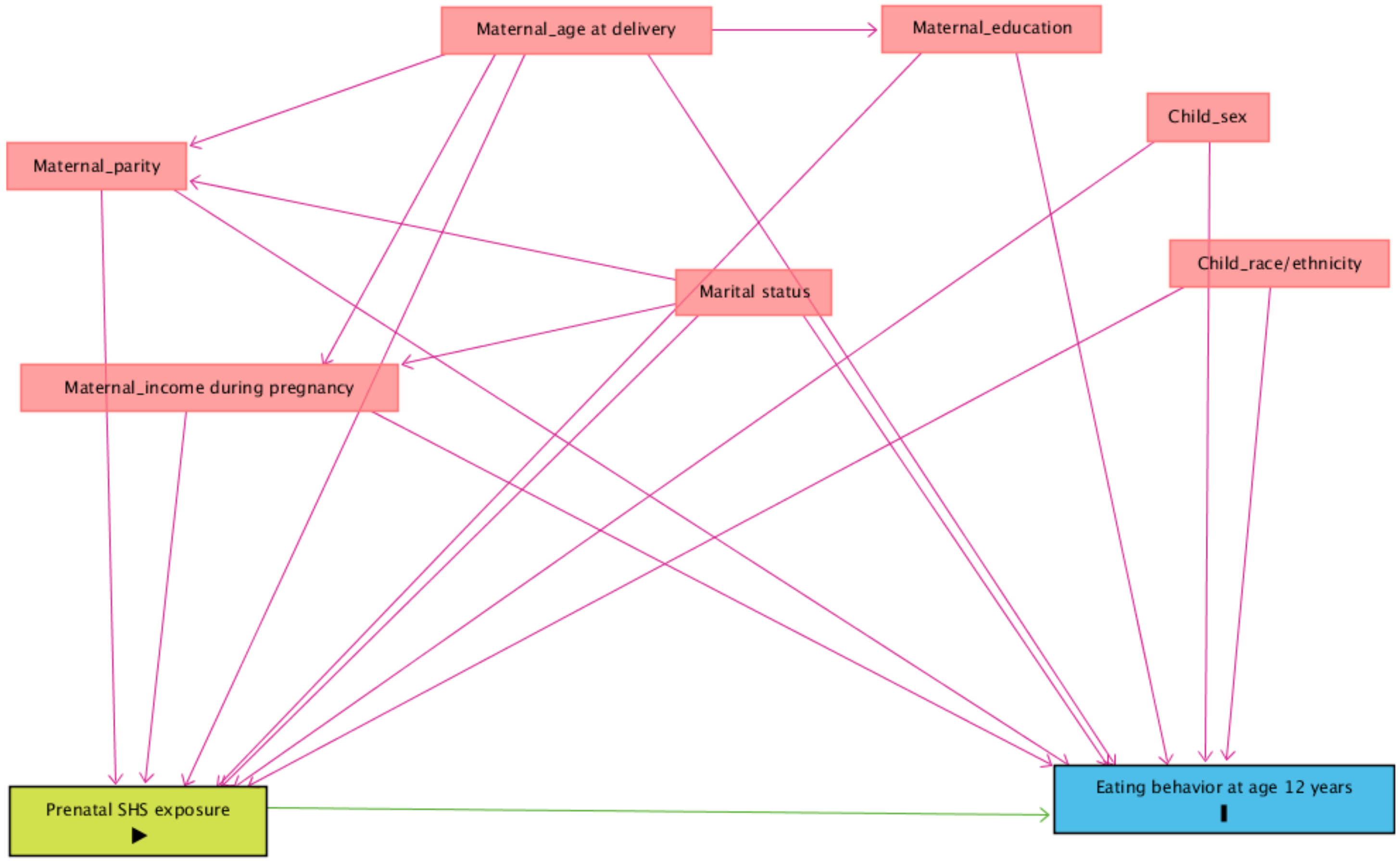

Figure S2. Directed acyclical graph of postnatal exposure to SHS, eating behaviors at age 12 years and potential confounders.

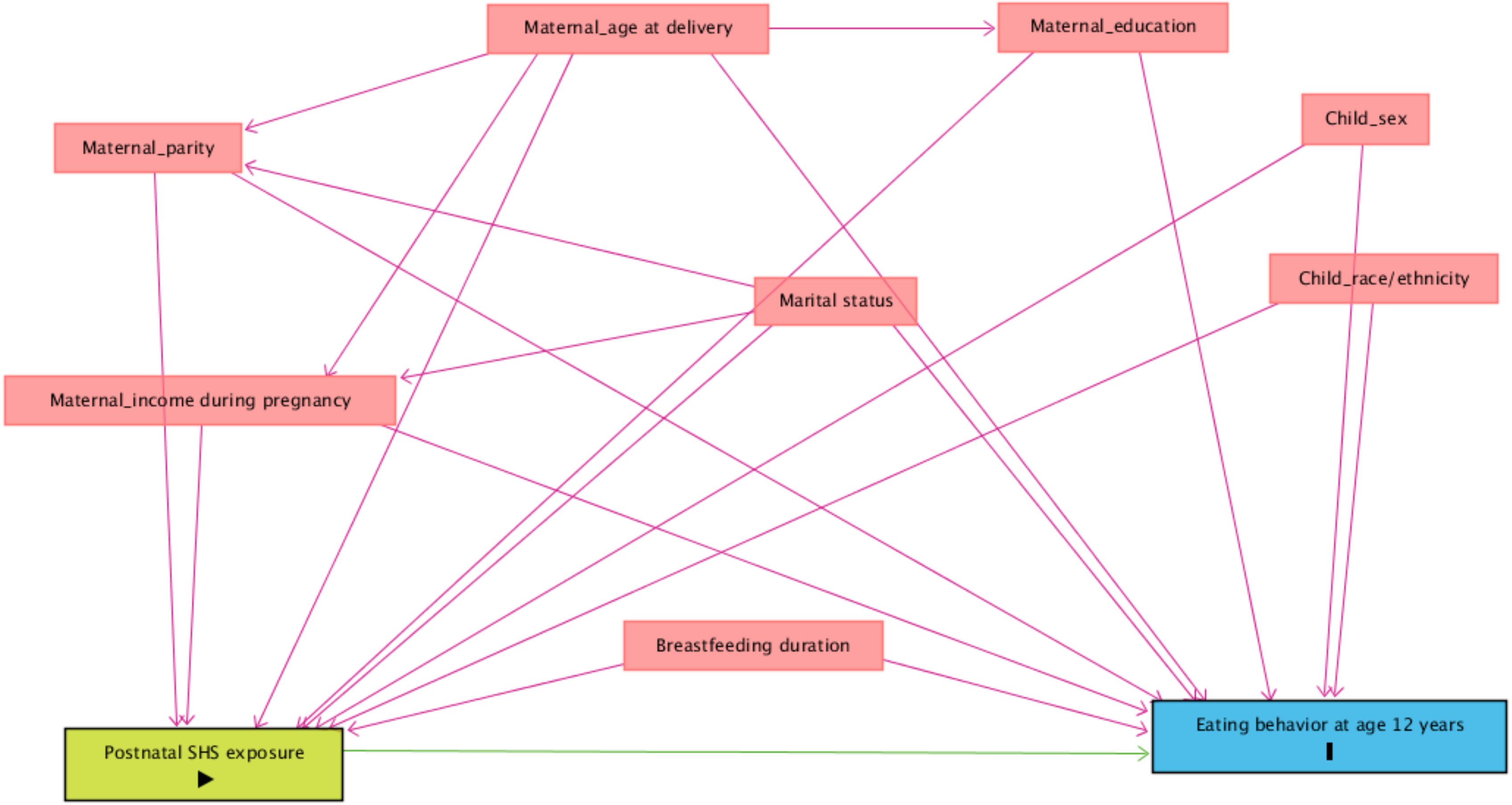

Figure S3. Violin plots of log_{10}-transformed prenatal and postnatal serum cotinine concentrations (ng/mL)[a] .

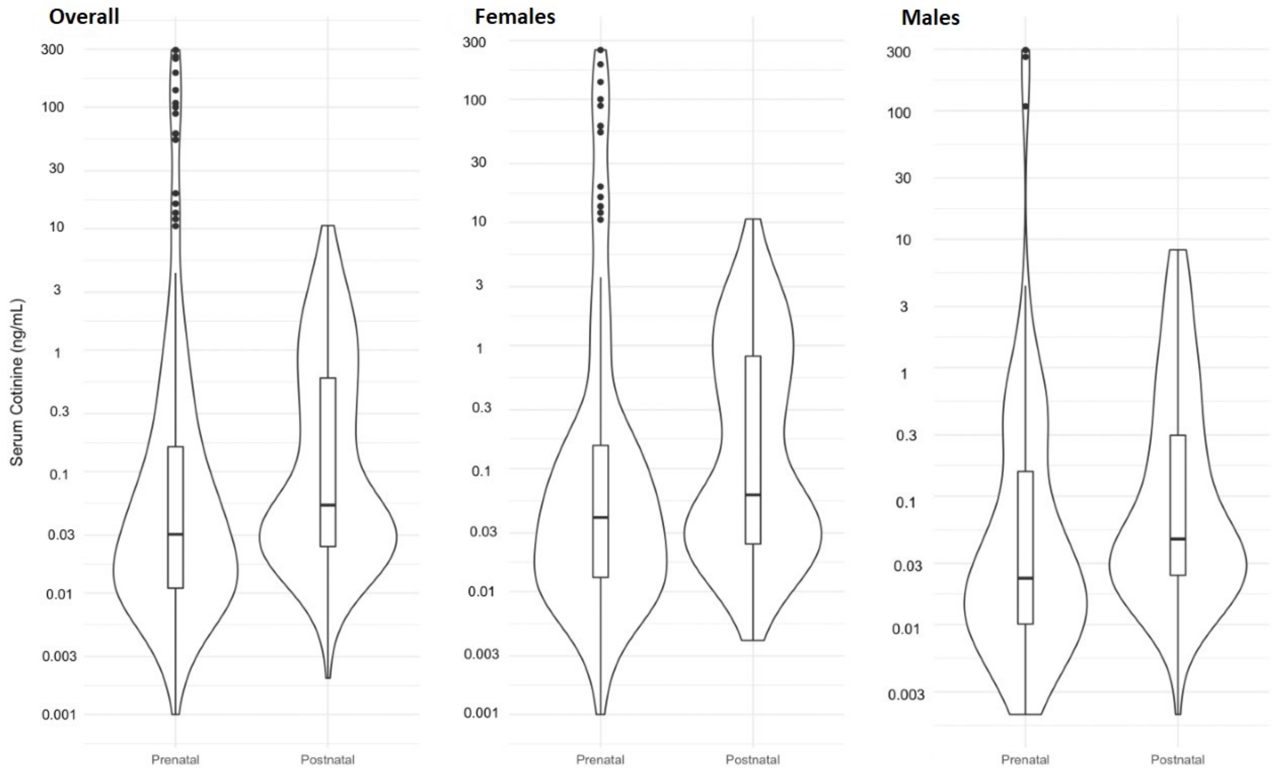

[a]The rectangle in the box plot (placed in the middle of each density curve) represents the 25, 50 (central dot) and 75 percentiles. Prenatal period includes maternal serum cotinine concentrations at 16 and 26 weeks of pregnancy. Postnatal period includes child's serum cotinine concentrations at ages 12, 24, 36 and 48 months.

Figure S4. Spearman correlation coefficients between adolescents' serum cotinine concentrations (N=207).

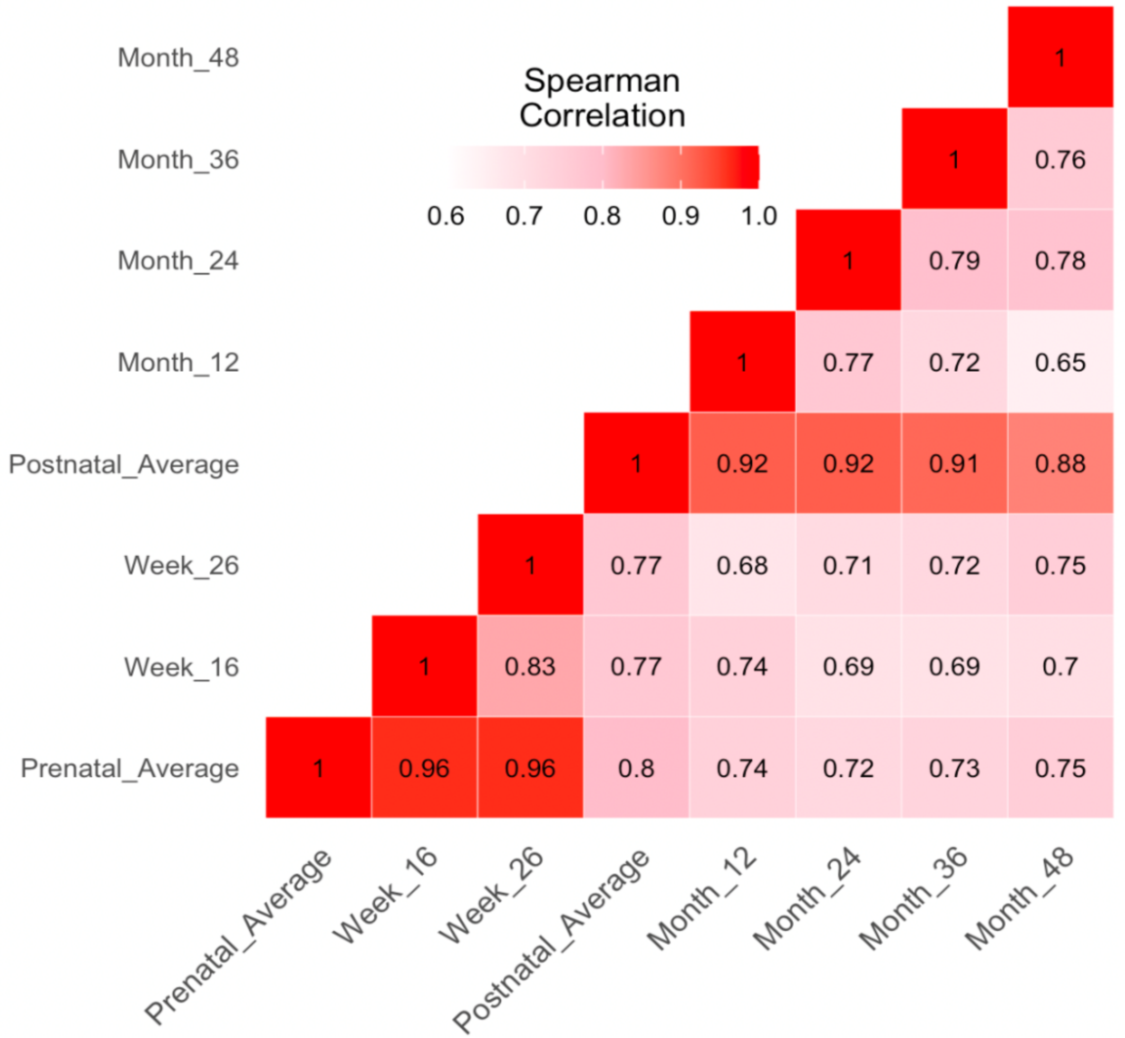

Supplement: Supplementary file 1 — Supplementary Material 1 [file 12940_2024_1076_MOESM1_ESM.pdf]
